# Not Just Nonspecific Factors: The Roles of Alliance and Expectancy in Treatment, and Their Neurobiological Underpinnings

**DOI:** 10.3389/fnbeh.2018.00293

**Published:** 2019-01-29

**Authors:** Sigal Zilcha-Mano, Steven P. Roose, Patrick J. Brown, Bret R. Rutherford

**Affiliations:** ^1^Department of Psychology, University of Haifa, Haifa, Israel; ^2^New York State Psychiatric Institute, Columbia University College of Physicians and Surgeons, New York, NY, United States

**Keywords:** alliance, expectancy, common factor, nonspecific factors, psychotherapy, mechanisms of change, process research

## Abstract

Therapeutic factors such as alliance and expectancy have been found to greatly affect treatment outcome in both psychotherapy and psychopharmacotherapy. Often, these factors are referred to as nonspecific because of their common roles across treatment modalities. Here we argue that conceptualizing such factors as nonspecific is not accurate at best, misleading at worst and may undermine treatment outcome across various modalities. We argue that alliance and expectancy contain both a trait-like common factor component and a state-like specific effect, and that it is clinically, conceptually and methodologically critical to disentangle the two. In other words, both alliance and expectancy may also function as active ingredients of treatment, leading to better outcome. We review the literature regarding the neurobiological underpinnings of alliance and of the expectancy effect, and suggest how future studies on the neurobiological basis of these effects can shed further light on the potentially distinct mechanisms of the trait-like and state-like components of each therapeutic factor.

One of the most debated questions in psychotherapy research is whether psychotherapies, psychopharmacotherapy and other treatments for mental health operate mainly through specific or nonspecific common factors (Mulder et al., 2017). The division of potential factors into specific and nonspecific has become a common framework for conceptualizing the factors affecting the process of therapeutic change. Theorists and researchers generally refer to specific effects as those factors that are described in treatment manuals and are considered specific to a psychotherapeutic orientation (e.g., cognitive restructuring in depression, exposure in anxiety disorders, or interpretations of transference) or the active chemical ingredients in a drug (e.g., increasing the extracellular level of the neurotransmitter serotonin by limiting its reabsorption into the presynaptic cell as with SSRIs). By contrast, nonspecific and common factors refer to those factors that are shared across most if not all forms of therapy. Typical examples of such factors are the therapeutic alliance between patients and their therapist or physician, and patients’ levels of expectancy regarding the process and outcome of treatment (Rosenzweig, 1936; Laska et al., 2014).

The division of factors into one of the two categories generally positions specific factors as the ones that are under the therapist’s control and need to be in the focus of therapist’s attention when seeking to improve treatment outcome. Nonspecific or common factors, by contrast, are those that everyone agrees are part of successful treatment, but at the same time are often taken for granted or considered to be factors that are outside of the therapist’s control. For example, the ability to form a strong alliance is often perceived strictly as a byproduct of the patients’ ability to form an adaptive relationship with others, and not something that the therapist can influence (DeRubeis et al., 2005). Given the division to specific vs. non-specific factors, it is not surprising that many psychotherapy research manuals describe techniques related to specific factors, and most therapist training focuses on such factors, and that in psychopharmacotherapy, most of the time and money allocated by pharmaceutical companies is to establish the mechanism of action of the drugs. Similarly, the knowledge derived from the division of factors into specific and nonspecific has produced few manuals that describe how to improve alliance, and little training has been devoted to this subject, especially in psychopharmacotherapy. Several notable exceptions in psychotherapy research include the work by Safran and Muran (2000) on training therapists in repairing alliance ruptures, and the work by Stiles and colleagues on therapists responsiveness to patient requirements and characteristics (Stiles, 2013; Kramer and Stiles, 2015). Similarly, despite the extensive knowledge produced by empirical studies regarding the important role of expectancy in treatment, no manual exists on techniques to boost expectancy. The scarce attention paid to the so-called nonspecific factors in manuals and training stands in contrast to the findings that they explain a significant amount of variance in treatment outcome. At least in the cases of alliance in psychotherapy and of expectancy in psychopharmacotherapy, meta-analyses and empirical studies suggest that they are stronger predictors of outcome than are specific psychotherapy techniques (Horvath et al., 2011 vs. Webb et al., 2010), and that they have a strong effect relative to the active effects of a drug (Rutherford and Roose, 2013).

We argue that treating therapeutic factors that have been found to be some of the stronger predictors of outcome as “nonspecific” greatly impairs the ability to fully understand their implications and realize their potential to bring about better therapeutic outcomes (Laska et al., 2014). We argue further that the distinction between common and specific factors is fundamentally problematic, and that each nonspecific factor may include both specific and nonspecific components. In other words, each such ingredient of the treatment may serve as a common facilitating environment and be deliberately used as an active ingredient of treatment, leading to better outcome. We support our argument using the cases of two factors commonly defined as nonspecific, alliance and expectancy, with examples from psychotherapy and psychopharmacotherapy. We present the available knowledge regarding the neurobiological basis of each, and suggest how the framework proposed here can be used to investigate potential distinct neurobiological mechanisms underpinning the specific and non-specific components of each therapeutic factor.

## The Roles of the Working Alliance in Treatment

### Common vs. Specific Roles of Alliance in Treatment

The relationship between the patient and the therapist or physician has been found to have a crucial effect on the success of any treatment, as has been demonstrated both indirectly, through meta-analyses testing the effect of the number of visits with the therapist or physician on outcome, and directly, focusing on explicit measures assessing the therapeutic relationships. Some of the indirect support for the importance of the therapeutic relationship is derived from studies and meta-analyses demonstrating that across psychotherapies (Falkenström et al., 2016), and even in antidepressant medication (ADM) treatment, the number of meetings with the therapist, which may represent opportunities for therapeutic interaction, affects treatment outcome. For example, meta-analyses suggest that in both placebo and ADM conditions more visits with the treating physician resulted in significantly greater symptom reduction (Rutherford et al., 2014). Although the effects were common across conditions, highlighting the common factor component of the interactions with the therapists, the effects also showed specificity, and were significantly more robust in the placebo condition. For example, it has been demonstrated that for patients receiving placebo, where no other active treatment is administered, the interactions with the physician may play a more active role than for patients receiving ADM. Among placebo recipients, between weeks 2 and 6, patients with weekly visits improved by 4.24 points on the Hamilton Rating Scale for Depression (HRSD; Hamilton, 1960), whereas those with one fewer visit improved by 3.33 points, and those with two fewer visits improved by 2.49 points (Posternak and Zimmerman, 2007). Additional visits explained approximately 50% of the symptom change observed between weeks 2 and 6 in patients receiving placebo. The magnitude of this effect was about 50% lower for participants receiving active medication.

A more recent meta-analysis further suggests that intensifying supportive care from 6 to 10 visits over 12 weeks resulted in a reduction of the average medication vs. placebo difference from 12.2% to 0.4% (Rutherford et al., 2014). Additional support to the specificity of the effect of visit frequency in placebo vs. ADM comes from another recent meta-analysis, showing that the increase in visit frequency in randomized controlled trials (RCTs) over the decades may, at least partly, account for the rise in placebo response at an average rate of 7% per decade over the past 30 years (Furukawa et al., 2016). Thus, visit frequency shows an effect across treatment modalities, supporting the common factor component of alliance. Moreover, the specificity of the effect in the placebo vs. medication conditions supports the specific factor component of alliance. This specific effect may be explained by additional visits providing additional opportunities for supportive empathic interactions with the physician (Rutherford and Roose, 2013). Indeed, studies suggest that the placebo effect is larger in a group receiving acupuncture treatment by a warm, empathic practitioner than in a group receiving treatment by a neutral practitioner (Kaptchuk et al., 2008; Kelley et al., 2009).

Decades of empirical research provided additional direct support of the importance of a strong therapeutic relationship for the success of treatment, both as a common and as a specific factor. These studies assessed the associations between measures of the therapeutic relationship, most commonly defined as the working alliance, and treatment outcome. The working alliance is commonly defined as the emotional bond established in the therapeutic dyad, and the agreement between the two about the goals of therapy and the tasks necessary to achieve them (Bordin, 1979; Hatcher and Barends, 2006). Meta-analyses have consistently demonstrated that stronger alliance is associated with better treatment outcome across treatment modalities, both in various psychotherapies (based on a data from more than 30,000 patients; Flückiger et al., 2018) and in psychopharmacotherapy (*N* = 1,065 patients; Totura et al., 2017).

Theoretical conceptualizations posit that the alliance plays a more active role in some treatments than in others (Safran and Muran, 2000; Castonguay et al., 2010), arguing, for example, that in cognitive behavioral treatments (CBTs) alliance serves the role of a nonspecific factor, enabling the effective use of various CBT techniques, whereas in alliance-focused treatment (AFT) it serves as a mechanism of change in itself. For decades, studies have failed to demonstrate that the extent to which alliance plays an active role in affecting treatment outcome differs by treatment modality. Only in recent years, with advances in trial design (notably, session-by-session measurement of the alliance) and in statistical methods to disentangle within- and between-patient variances (Wang and Maxwell, 2015)^1^, has it become possible to thoroughly and systematically examine the distinct roles that alliance plays in treatment success, and to differentiate between the roles of alliance as a common and as a specific factor.

Recent studies demonstrate the importance of separating the trait-like and state-like components of the alliance, each of which play a distinct role in treatment (Zilcha-Mano, 2016, 2017). The trait-like component refers to the way in which trait-like characteristics of the patients, such as their ability to form satisfying relationships with others, affect their ability to create, with their therapist, the environment required to conduct any effective treatment. The trait-like component of alliance is a product of the patients’ (and the therapists’) trait-like characteristics, such as attachment orientation. Some individuals have better trait-like capacity to form strong and satisfying relationships with significant others. Empirical studies suggest that these capabilities affect their tendency to create a strong helping relationship with their therapist (Barber et al., 2002; Haggerty et al., 2009; Zilcha-Mano et al., 2014, 2015a), which is the environment facilitating the conduct of any effective treatment. Indeed, patients with such adaptive trait-like characteristics improve more following treatment than do patients without such characteristics (Hoffart et al., 2013; Zilcha-Mano and Errázuriz, 2015; Zilcha-Mano et al., 2015c). This component, however, is not sufficient in itself to induce change, and it is mainly a product of other trait-like characteristics of the patient.

By contrast, the state-like component of alliance serves as a mechanism of change in itself, such that changes in this component of the alliance are the cause of subsequent symptomatic change (Zilcha-Mano, 2017). The state-like component represents the role of alliance as an active ingredient, capable of inducing therapeutic change in itself. During the process of therapeutic change, the patient develops abilities to form a strong and satisfactory alliance with the therapist, resulting in better outcomes. Empirical studies suggest that state-like changes in alliance significantly predict subsequent treatment outcome over the course of treatment (Falkenström et al., 2013; Zilcha-Mano and Errázuriz, 2015; Zilcha-Mano et al., 2015c), supporting their role in bringing about therapeutic change. The state-like component may function as an active ingredient, whereas the trait-like component may act as a common/non-specific one.

Recent empirical studies have demonstrated that treatments differ in their state-like effect of alliance on outcome, but not in their trait-like effect (Zilcha-Mano, 2016, 2017), further supporting the distinction between the portion of the alliance that serves as a common factor across treatment, and the portion that has a specific effect in treatments in which the alliance is expected to be an active ingredient. In these studies, the state-like component was found to have a greater effect on outcome in treatments in which alliance is conceptualized as a mechanism of change, such as in AFT, as opposed to treatments in which it is conceptualized as a common factor, such as in CBT (Zilcha-Mano et al., 2016). The state-like component was also found to have a stronger effect on outcome in placebo than in the ADM condition (Zilcha-Mano et al., 2015b). Moreover, the state-like component was found to have a stronger effect for patients with relatively poor capabilities to form satisfying relationships with others (Zilcha-Mano and Errázuriz, 2017), such that the state-like alliance had a stronger effect on outcome for those with more vs. less interpersonal problem. Taken together, studies suggest that state-like alliance may have a specific effect for those who have more interpersonal problems, and in treatment in which the alliance is conceptualized as an active ingredient. Recent studies further suggest that the magnitude of the state-like effect of alliance on outcome can be manipulated by providing therapists with continual feedback on alliance, as rated by their patients, throughout the course of treatment (Zilcha-Mano and Errázuriz, 2015). Taken together, these studies support both a common factor component of alliance and a clear specific component, and they demonstrate that the state-like component is not merely a nonspecific factor, but rather can be manipulated and used for treatment success.

### Neurobiological Underpinning of the Working Alliance

Studies are only now starting to illuminate the neurobiological basis of the effect of alliance. Most of this literature is still tentative, referring to neurobiological mechanisms that have the potential to serve as markers of the alliance, but were never explicitly tested as such. Some of the most promising paths include the literature on “mirror neurons”, originally discovered in the premotor cortex of monkeys, which are activated when an individual observes an activity, in a similar way to when performing it (Gallese et al., 1996). Activation of these areas was found to be related to empathy, and deficits were linked to disorders characterized by interpersonal impairments, such as autism (Dapretto et al., 2006; Iacoboni and Dapretto, 2006; Cattaneo and Rizzolatti, 2009; Le Bel et al., 2009).

Another promising path involves the role of hormones, such as oxytocin and cortisol, as potential bio-markers. It has been suggested that the effects of comforting interactions with a therapist on outcome, and their role in regulating stress and inflammation, may be mediated in part by the release of oxytocin (Brown and Brown, 2015). Administration of oxytocin has been shown to regulate stress at a variety of levels, including decreasing blood pressure and the stress hormone cortisol, as well as increasing progesterone, a regulatory hormone that restores GABAergic tone following activation of the hypothalamic-pituitary-adrenal axis (Childs et al., 2010). To our knowledge, only one study to date has examined empirically the biomarkers of alliance (Zilcha-Mano et al., 2018b). The study focused on oxytocin and found converging associations between both self-reported alliance and behavioral coding of alliance by external coders, and changes in oxytocin during psychotherapy sessions throughout treatment. These associations were found only after disentangling state-like and trait-like effects, further supporting the importance of untangling the two components. Future studies can use the empirical data collected on the two distinct components of alliance, the state-like and the trait-like, to investigate potential distinct neurobiological markers of each component. It may be the case that the same neurobiological systems are involved in both but in different ways, or that different systems are active in each one. For example, greater increase in oxytocin during the sessions may be found in conditions in which the specific component of alliance is active. Similarly, other agents, such as cortisol, may be at work whenever the common factor component of alliance is dominant. For example, in sessions which include extinction-based interventions, superior therapeutic gains were found when cortisol levels where higher than lower (Meuret et al., 2015).

## The Roles of Expectancy in Treatment

### Common vs. Specific Roles of Expectancy in Treatment

Expectancy refers to the patients’ beliefs about whether and how much they expect to improve as the consequence of the treatment (Rutherford et al., 2017b). Expectancy can be conceptualized as including both a facilitating component, which is common across treatments, and an active therapeutic component (Zilcha-Mano et al., 2018a). The trait-like component refers to individual differences between patients in their general tendency to show high levels of expectancy, which is a product of the patients’ other characteristics, such as degree of general optimism vs. pessimism, perceived locus of control, and other psychological factors. By contrast, the state-like component refers to the changes in expectancy within individual patients over the course of treatment, which may be related to events in the treatment process. The vast majority of studies have focused on the trait-like component of expectancy, arguing that it can serve as a common factor across therapeutic modalities (Kirsch, 1990; Rutherford and Roose, 2013). This claim has been supported by accumulating findings, demonstrating the effect of trait-like expectancy across treatment modalities (Constantino, 2012). For example, a secondary analysis based on data collected in the Treatment of Depression Collaborative Research Program showed that higher levels of expectancy at baseline were associated with higher likelihood of complete response, and lower level of depression post-treatment across all four treatment conditions: CBT, interpersonal psychotherapy (IPT), imipramine with clinical management (CM) and placebo with CM (Sotsky et al., 1991). A recent meta-analysis of the association between patients’ expectancy and post-treatment outcome across a variety of psychotherapies and clinical contexts further supports the importance of expectancy across treatment modalities (Constantino et al., 2018): based on the data of 12,722 patients across 81 independent samples, a small but significant effect emerged, according to which higher levels of expectancy were associated with better treatment outcome.

Although most of the literature on expectancy has focused on the common factor role of trait-like expectancy, there are promising findings to support also a specific role for expectancy, especially in the above-mentioned meta-analysis and in the latest empirical literature on expectancy. In addition to demonstrating the role of expectancy as a common factor across treatment modalities, the meta-analysis also supports the specificity of the effect, such that some patients may benefit more than others from increased expectancy for the success of treatment (Constantino et al., 2018). Specifically, the effect of expectancy on outcome is weaker as patients age. Similar findings regarding the specificity of the expectancy effect in younger vs. older adults have been demonstrated in psychopharmacotherapy as well (Rutherford et al., 2017b; see also Rutherford et al., 2017a).

Studies further suggest that expectancy may increase during treatment and that such increases may affect treatment outcome. Higher levels of expectancy were found to follow more competent use of techniques (for example, in delivering CBT for generalized anxiety disorder), and the higher levels of expectancy were in turn associated with better post-treatment outcome (Westra et al., 2011). In another study, stronger early alliance was related to higher patient expectancy, which in turn was associated with fewer post-treatment interpersonal problems (Vîslă et al., 2018). Although these studies attest to the potential promising effect of state-like expectancy, they did not manipulate expectancy, nor did they examine how expectancy changes over treatment. Because expectancy is generally perceived as a common nonspecific factor and not as a factor that includes a state-like component that can be increased during treatment, almost all studies on expectancy assessed it only at baseline or in early treatment (Constantino et al., 2018). Yet, several studies have focused directly on state-like expectancy and demonstrated its effect on treatment outcome. Recently, a prospective randomized trial manipulated expectancy and tested the effect of such manipulation on outcome. The study showed that increasing pre-treatment expectancy levels by manipulating patients’ chances of receiving ADM vs. placebo (increasing it from 50% to 100% probability) resulted in greater reduction in symptoms (Rutherford et al., 2013, 2017b). These findings are further supported by a series of meta-analyses demonstrating that patients who know they are receiving medication, that is, those in comparator or open trials, show significantly greater medication response (mean of 15% higher) than those receiving medication as part of a placebo-controlled trial, who do not know whether they received medication or placebo (Rutherford et al., 2009, 2017b). Consistent with these results, in their meta-analysis, Papakostas and Fava (2009) reported that the probability of receiving placebo in a clinical trial was negatively correlated with antidepressant and placebo response, such that for each 10% increase in the probability of receiving placebo, the probability of antidepressant response decreased 1.8% and the probability of placebo response decreased 2.6%.

The studies on the effects of pre-treatment expectancy manipulation on outcome shed important light on the potential for augmenting expectancy as a tool for improving treatment efficacy. This literature, however, is limited to pre-treatment expectancy, and does not account for changes in expectancy during treatment. A recent study from our group focused on the state-like component of expectancy and showed that state-like changes in expectancy indeed occur during the course of treatment, both in the ADM and the placebo conditions (Zilcha-Mano et al., 2018a). The study further suggested that state-like changes in expectancy are not merely a byproduct of changes in symptoms, but rather predicted subsequent changes in symptoms. Taken together, the findings support significant effects of both a trait-like, non-specific common factor component and a state-like specific active ingredient component, of expectancy on outcome.

### Neurobiological Underpinning of Expectancy

Similarly to the literature on alliance, studies are only now starting to cover the neurobiological basis of the effect of expectancy. Most of this literature refers to neurobiological mechanisms that have the potential to serve as markers of expectancy, based on their roles in emotional appraisal and in placebo analgesia. Accumulating studies have established that the prefrontal cortex (PFC) is critical to the cognitive regulation of emotion, particularly the dorsolateral, ventrolateral and ventromedial prefrontal cortices (DLPFC, VLPFC and VMPFC; Ochsner and Gross, 2005). PFC regions reciprocally connect with subcortical areas such as the amygdala, nucleus accumbens (NAcc) and insula, which are important for appraising the aversive or rewarding properties of stimuli (O’Doherty et al., 2002). Focusing on placebo effect in major depression, studies demonstrate the important roles of prefrontal and striatal regions as well as of the opioid system (e.g., Peciña et al., 2014).

It has been suggested that a PFC-amygdala pathway underlies a negative appraisal process, leading to the generation of negative emotional responses to stimuli (Wager et al., 2008). In studies of placebo analgesia, expecting pain relief before a painful stimulus leads to increased activation in the DLPFC/VMPFC, decreased activation of the amygdala and insular regions, and increases in NAcc activation (Wager et al., 2004). These findings suggest that expectancy may lead to improvement in depressive symptoms by reversing depressed patients’ mood-congruent processing bias toward negative emotions, and ameliorating impaired reward functioning (Chiu and Deldin, 2007; Vallance, 2007). There is evidence to suggest that antidepressant treatments may indeed function by normalizing these pathological increases in limbic activity (Fu et al., 2004; Arce et al., 2008). Recent findings by our group suggest that that manipulation aimed at raising expectancy in patients with MDD reduced activation in the left amygdala, which in turn resulted in a more effective treatment.

An ongoing trial by our group seeks to disentangle the trait-like and state-like components of expectancy and to investigate their distinct potential neurobiological underpinnings. For example, based on the accumulating literature, it is possible to cautiously suggest that white matter hyperintensities (WMH) may underlie the effect of the state-like component of expectancy. WMH have been associated with poor response to antidepressants (Simpson et al., 1997; O’Brien et al., 1998). According to the vascular depression model, vascular lesions in deep white matter tracts disconnect prefrontal antidepressant response in depressed patients, so that WMH results obtained with serotonergic medications are less efficacious in the presence of this structural brain pathology. WMH burden was related to especially high limbic hyperactivity in response to emotional face stimuli (Aizenstein et al., 2011). WMH damage is assumed to interrupt the neural circuitry underlying expectancy-based placebo effects. Such damage is not expected to interfere with the formation of expectancies, therefore the common factor component is not expected to be affected. Rather, WMH damage is expected to be related to difficulty updating and maintaining appropriate treatment expectancies in response to new information regarding the treatment being received. The vascular damage to frontostriatal tracts may limit the top-down modulation of limbic and striatal structures necessary for depressive symptom change. Thus, the specificity of WMH as an underlying neurobiological mechanism for state-like but not trait-like expectancy can be expected.

Additional support for the state-like component of expectancy comes from studies demonstrating that the update of expectation over time may influence the response to placebo in the treatment for pain (Peciña et al., 2014; Schafer et al., 2018). Specifically, the discrepancy between expectations and subjectively rated effectiveness was found to be associated with placebo analgesic responses, and with the activation of regional m-opioid neurotransmission in a substantial number of regions implicated in opioid-mediated antinociception. The largest placebo responses were observed in those with low expectations and high subjective effectiveness (Peciña et al., 2014).

## Concluding Remarks

The most recent studies on both expectancy and alliance suggest that these two central examples of common, nonspecific factors contain both common, trait-like effects across studies, and specific effects, which can be manipulated to affect treatment outcome. Separating trait-like and state-like components is of great importance for conceptual, clinical and methodological reasons. Conceptually, separating trait-like and state-like components is critical to move toward a comprehensive perspective that replaces the one-dimensional, partial understanding of common factors that is prevalent today. Clinically, the separation may provide additional tools for therapists to improve treatment outcome. Expanding the therapist’s repertoire of tools is essential for moving toward personalized medicine, which endeavors to make use of the most beneficial individually-tailored tools in the treatment of each patient. For example, developing a manual to improve treatment expectancy may be beneficial across treatment modalities (the common factor expectancy component), and especially beneficial with certain populations (the state-like expectancy component). Such information can be particularly valuable in treatment selection processes with populations such as the elderly, which showed clear deficits in the ability to benefit from manipulations aimed at boosting expectancy (Rutherford et al., 2017a,b). The methodological literature also demonstrates how crucial it is to disentangle these two components if one seeks to explore causal relationships during treatment (Curran and Bauer, 2011; Wang and Maxwell, 2015). Our argument for disentangling the trait-like and state-like components of what was previously referred to as “nonspecific” factors is also consistent with progress toward identifying commonalities between treatments and at the same time identifying the uniqueness in each. For example, common patterns of symptom reduction (such as sudden gains) have been identified across treatment modalities, but their precursors were found to be unique and specific for each treatment (Tang and DeRubeis, 1999; Andrusyna et al., 2006).

As we demonstrated using the cases of expectancy and alliance, common factors are associated with therapeutic outcome across treatments. The strength of this effect, however, is not common (the effect may be greater in some treatments and in some populations than in others, such as in younger vs. older individuals), and can even be manipulated. Labeling these therapeutic ingredients as nonspecific may result in underestimating their role and treating them as a minor, unchangeable part of treatment. Although we based our arguments on the most central factors identified in the literature as common nonspecific factors, we believe that implementing the suggested framework for differentiating trait-like and state-like effects can be instrumental in revealing the components of many of the constructs that have been referred to as nonspecific factors. Note further that although we discussed alliance and expectancy separately, they are not unrelated but rather interdependent constructs (Vîslă et al., 2018). For example, it has been suggested that higher patient pre- or early-treatment expectancy is related to stronger alliance, which in turn correlates with better outcomes (Yoo et al., 2014; Vîslă et al., 2018).

It is of great importance to establish neurobiological signatures for the effects of therapeutic factors in treatments, especially to examine whether the state-like vs. trait-like components of each factor are based on distinct neurobiological signatures. Such signatures may help demonstrate the distinct effect of each component in treatment. Neurobiological markers have also the potential to complement and improve the accuracy of clinical assessment of the process and outcome of treatment. Future studies on state-like and trait-like components of therapeutic factors will be instrumental in designing therapeutic interventions that make use of the heterogeneity of expectancy and alliance effects. It is reasonable to expect that not all patients will derive the same benefits from each therapeutic factor. Therefore, such studies are critical for progress toward personalized treatment and for producing actionable, prescriptive information about which interventions are best suited for which patients.

## Author Contributions

All authors contributed equally to the conceptualization of the idea and the writing of the manuscript.

## Conflict of Interest Statement

The authors declare that the research was conducted in the absence of any commercial or financial relationships that could be construed as a potential conflict of interest.

## Footnotes

^1^State-like and trait-like components can be disentangled by untangling between- and within-patient variance in the alliance and its effect on outcome. Several methods are available for disentangling between- and within-patient effects in longitudinal data, including centering and detrending of the variable. Centering is the statistical operation of subtracting from each individual’s measurements the mean of that individual’s measurements. Detrending is the statistical operation of removing the time trend, in addition to centering. Using detrending methods, it is possible to control for the effect of time while examining the relation between the dependent and independent variables. For more information about the two methods, see Wang and Maxwell (2015).
